# Possibility of Using Conventional Computed Tomography Features and Histogram Texture Analysis Parameters as Imaging Biomarkers for Preoperative Prediction of High-Risk Gastrointestinal Stromal Tumors of the Stomach

**DOI:** 10.3390/cancers15245840

**Published:** 2023-12-14

**Authors:** Milica Mitrovic Jovanovic, Aleksandra Djuric Stefanovic, Dimitrije Sarac, Jelena Kovac, Aleksandra Jankovic, Dusan J. Saponjski, Boris Tadic, Milena Kostadinovic, Milan Veselinovic, Vladimir Sljukic, Ognjan Skrobic, Marjan Micev, Dragan Masulovic, Predrag Pesko, Keramatollah Ebrahimi

**Affiliations:** 1 Center for Radiology and Magnetic Resonance Imaging, University Clinical Centre of Serbia, Pasterova No. 2, 11000 Belgrade, Serbia; milica.mitrovic-jovanovic@med.bg.ac.rs (M.M.J.);; 2 Department for Radiology, Faculty of Medicine, University of Belgrade, Dr Subotica No. 8, 11000 Belgrade, Serbia; 3 Department for HBP Surgery, Clinic for Digestive Surgery, University Clinical Centre of Serbia, Koste Todorovica Street, No. 6, 11000 Belgrade, Serbia; 4 Department for Surgery, Faculty of Medicine, University of Belgrade, Dr Subotica No. 8, 11000 Belgrade, Serbia; 5 Center for Physical Medicine and Rehabilitation, University Clinical Centre of Serbia, Pasterova Street, No. 2, 11000 Beograd, Serbia; 6 Department of Stomach and Esophageal Surgery, Clinic for Digestive Surgery, University Clinical Centre of Serbia, Koste Todorovica Street No. 6, 11000 Belgrade, Serbia; 7 Department for Pathology, Clinic for Digestive Surgery, University Clinical Centre of Serbia, Koste Todorovica Street, No. 6, 11000 Belgrade, Serbia

**Keywords:** gastrointestinal stromal tumor (GIST), multidetector computed tomography (MDCT), texture analysis, metastatic risk

## Abstract

**Simple Summary:**

Gastrointestinal stromal tumors are the most common mesenchymal tumors that can have a malignant character. Definitive diagnosis is obtained by pathohistological and immunohistochemical analysis of the resected tumor. Preoperative stratification of metastatic risk using non-invasive imaging methods would be of great importance in the selection of patients with high-risk GIST and the application of neoadjuvant target therapy. This could enable tumor shrinkage, avoiding multivisceral resections and reducing the risk of tumor rupture. It also could provide better long-term outcomes, including increased overall survival rates, by optimizing surgical resection and systemic control of the disease. Evaluation of the morphological characteristics of the tumor obtained by computed tomography examination as well as the histogram parameters of the textural analysis of tumor tissue may improve the preoperative prediction of the metastatic risk of GIST. Texture analysis is part of the growing field of radiomics, with significant contributions to oncology so far.

**Abstract:**

Background: The objective of this study is to determine the morphological computed tomography features of the tumor and texture analysis parameters, which may be a useful diagnostic tool for the preoperative prediction of high-risk gastrointestinal stromal tumors (HR GISTs). Methods: This is a prospective cohort study that was carried out in the period from 2019 to 2022. The study included 79 patients who underwent CT examination, texture analysis, surgical resection of a lesion that was suspicious for GIST as well as pathohistological and immunohistochemical analysis. Results: Textural analysis pointed out min norm (*p* = 0.032) as a histogram parameter that significantly differed between HR and LR GISTs, while min norm (*p* = 0.007), skewness (*p* = 0.035) and kurtosis (*p* = 0.003) showed significant differences between high-grade and low-grade tumors. Univariate regression analysis identified tumor diameter, margin appearance, growth pattern, lesion shape, structure, mucosal continuity, enlarged peri- and intra-tumoral feeding or draining vessel (EFDV) and max norm as significant predictive factors for HR GISTs. Interrupted mucosa (*p* < 0.001) and presence of EFDV (*p* < 0.001) were obtained by multivariate regression analysis as independent predictive factors of high-risk GISTs with an AUC of 0.878 (CI: 0.797–0.959), sensitivity of 94%, specificity of 77% and accuracy of 88%. Conclusion: This result shows that morphological CT features of GIST are of great importance in the prediction of non-invasive preoperative metastatic risk. The incorporation of texture analysis into basic imaging protocols may further improve the preoperative assessment of risk stratification.

## 1. Introduction

Gastrointestinal stromal tumors (GISTs) are relatively rare mesenchymal tumors with a potentially malignant and aggressive behavior [1]. They can occur anywhere in the digestive tract, but are most commonly localized in the stomach and small intestine [2]. These tumors tend to spread and metastasize. As they are often detected at an advanced stage, they can pose a serious challenge for management. Surgery is the only curative treatment, and recently, the minimally invasive approach has proven to be feasible and safe [3].

Although these tumors do not have the same biological behavior, any GIST should be considered potentially malignant [3]. The location and size of the tumor are important factors that determine the modality of GIST treatment. Regardless of the size of the tumor, complete removal in challenging locations sometimes requires extensive, risky and mutilating surgery associated with functional disability or morbidity. In such cases, especially when the tumor is small, clinical guidelines generally recommend only follow-up [4,5].

The fact that a GIST is small does not exclude the possibility that its proliferative activity may be aggressive [6]. Tumor biopsy is the best way to obtain tissue samples for subsequent pathological diagnosis. However, it is associated with possible complications such as tumor rupture or bleeding, and the results are often inconclusive. Most GIST guidelines for the surveillance of small lesions recommend initial follow-up by EUS. The Japanese guidelines point out that GISTs may be potentially aggressive if the tumors show growth features ulceration or irregular margins at follow-up [5]. The NCCN sarcoma guidelines also recommend that a small tumor that has high-risk features should be removed, while others that do not have these features can be followed by EUS [7]. However, manu studies have showen that extrinsic or exophytic tumor growth may be missed by these examinations. In such situations, new or additional biomarkers would make an important contribution to deciding on the optimal treatment modality.

Given the potential benefits of and research on neoadjuvant therapy for GIST, preoperative risk stratification may be of particular importance. Preoperative administration of tyrosine kinase inhibitors could enable tumor shrinkage and reduce the risk of tumor rupture [8]. It also could provide better long-term outcomes, including increased overall survival rates, by optimizing the systemic control of the disease [9]. Two comparative systems are most commonly used in clinical practice: the Armed Forces Institute of Pathology (Miettinen’s) criteria and the National Institutes of Health (NIH) classification [10,11]. Both systems use the mitotic index as an important factor in the assessment of tumor aggressiveness. According to the Miettinen criteria, the risk of recurrence or metastasis in 2 to 5 cm gastric GISTs mainly depends on their mitotic activity. Studies have shown that grading based on mitotic count is not accurate in regular biopsy [12]. Considering the above facts, the neoadjuvant strategy for GISTs may complicate the selection of suitable patients.

The most commonly used diagnostic modality for the diagnosis and staging of these tumors is computed tomography (CT). Many studies have demonstrated a correlation of certain morphologic CT features of GISTs with a high metastatic risk, especially the diameter of the lesion [13,14,15,16]. CT texture analysis (CTTA) is a relatively new postprocessing imaging tool used to assess the heterogeneity of tumor tissue [17]. It has proven to be very useful in differentiating diagnosis, stratifying the grade and risk of different tumors, assessing prognosis and predicting the response to the implemented therapy [17,18,19,20].

The aim of this study is to evaluate the diagnostic value of the morphologic parameters of conventional CT diagnostics and the histogram parameters of texture analysis in the non-invasive, preoperative assessment of the metastatic potential of GISTs in correlation with macropathological and pathohistological findings, especially with the mitotic index, as a gold standard. Preoperative diagnosis of high-risk GISTs could facilitate decisions on further treatment protocol in patients in whom tumor localization requires multivisceral or extensive surgery. The use of neoadjuvant therapy with tyrosine kinase inhibitors in patients with HR GISTs would lead to tumor dimension reduction, thus effectively improving the resection rate of surgery. Further, it would also reduce the risk of tumor recurrence and lead to better prognosis of the disease [9].

## 2. Materials and Methods

### 2.1. Patients

Seventy-nine patients who underwent a CT diagnostic protocol followed by surgery during the period from 2019 to 2022 were included in this prospective research. Criteria for inclusion in the study were as follows: (1) clinically suspected GIST as mainly submucosal gastric lesion; (2) abdominal CT exam according to a dual-phase protocol; (3) no more than 20 days between CT examination and surgery. Criteria for exclusion from the study were as follows: (1) extra-gastric localization of GIST, (2) histopathological findings suggestive of other stomach tumors, (3) patients whose CT exam was of poor technical quality without the possibility of further processing and (4) more than 20 days from the performed CT examination to the surgical resection of the tumor. Gastric resections such as total and subtotal gastrectomy or wedge resection or tumor enucleation were performed in all patients with histopathological and immunohistochemical analysis of the resected tumor. Further, the tumors were staged using the American Joint Committee on Tumor/Lymph Node/Carcinoma Metastases (TNM) classification (8th edition) [21]. According to the TNM supplemented with AFIP classification, patients were divided into two groups, low-risk (LR) and high-risk (HR) [22].

Our research was permitted by the Ethical Committee of the Faculty of Medicine, University of Belgrade, and written informed consent was obtained from all patients.

### 2.2. Abdominal CT Examination

CT diagnosis was performed on a multidetector CT (MDCT) machine with 64 rows of detectors (Aquuilion One, Toshiba Medical Systems, Ottawa, Japan). Immediately before the examination, the patients were given 250–500 mL of water “per os” (as a negative contrast), in order to adequately distend the stomach.

Abdominal CT examination was performed as standard after iv. administration of 60–100 mL of iodinated contrast (1–1.5 mL/kg body weight), in the arterial and portal venous phase.

### 2.3. Abdominal CT Scan Analysis

**Figure 1:**
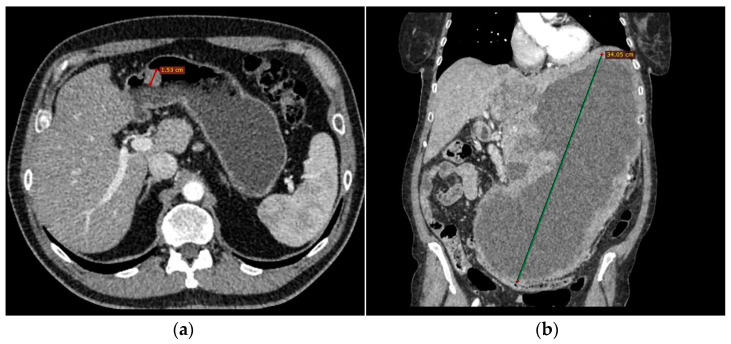
The lowest tumor diameter was 15 mm (LR GIST) in the pyloric stomach region (**a**) and the largest lesion measured 340 mm (HR GIST) (**b**). LR GIST shows a predominantly round shape, well-defined margins and a homogenous, solid appearance and intact mucosa (**a**). Notable difference in tumor structure with massive cystic degeneration, irregular shape and exophytic growth pattern in HR tumor (**b**).

**Figure 2:**
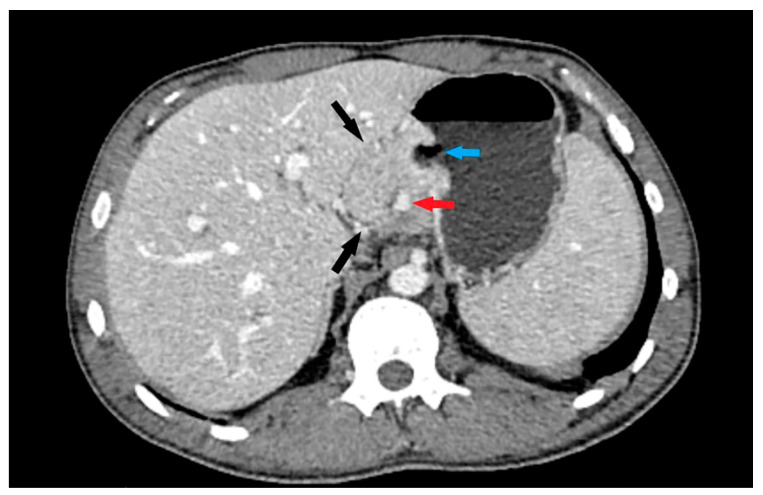
Axial CT scan shows exophytic tumor growth into gastro-hepatic ligament (black arrows) with presence of intralesional vascular structures (red arrow) and mucosal defect: umbilication (blue arrow). Tumor involves corpus region of the stomach.

**Figure 3:**
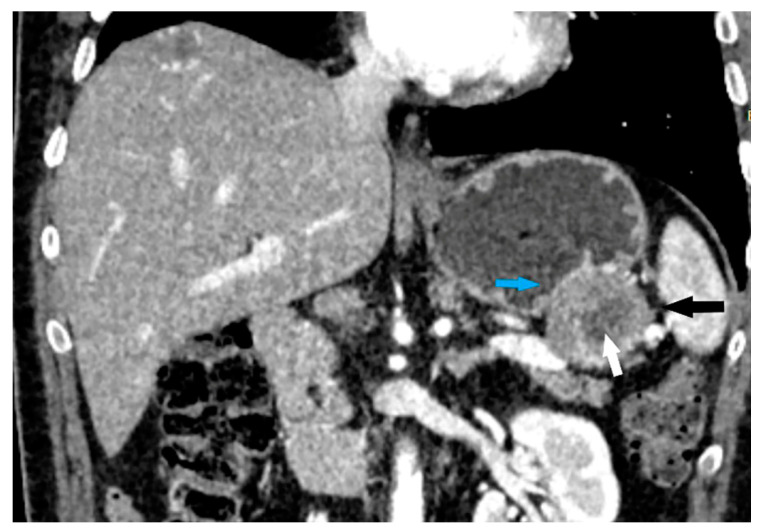
CT scan coronal view clearly depicts oval, clearly demarcated lesion (black arrow) covered by intact mucosa (blue arrow) with partially necrotic structure (white arrow) and weak postcontrast opacification of solid part of the lesion.

**Figure 4:**
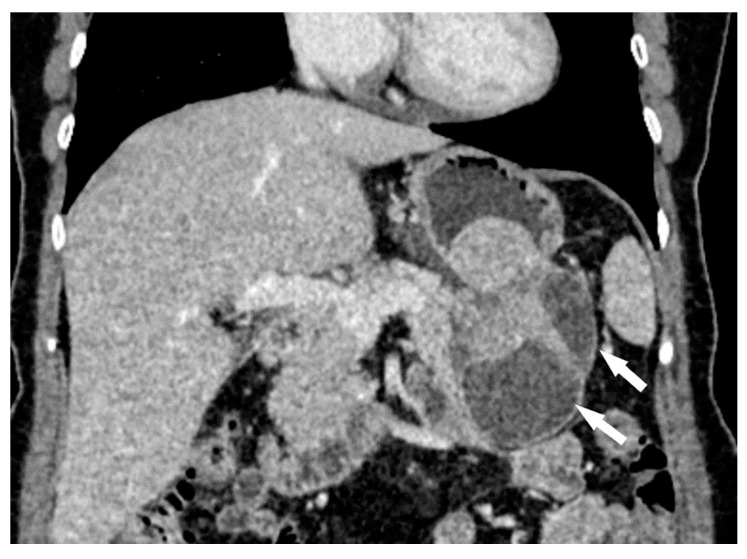
Abdominal CT exam coronal view shows irregular tumor shape with presence of cystic structural changes (white arrow). The tumor corresponds to HR GIST.

**Figure 5:**
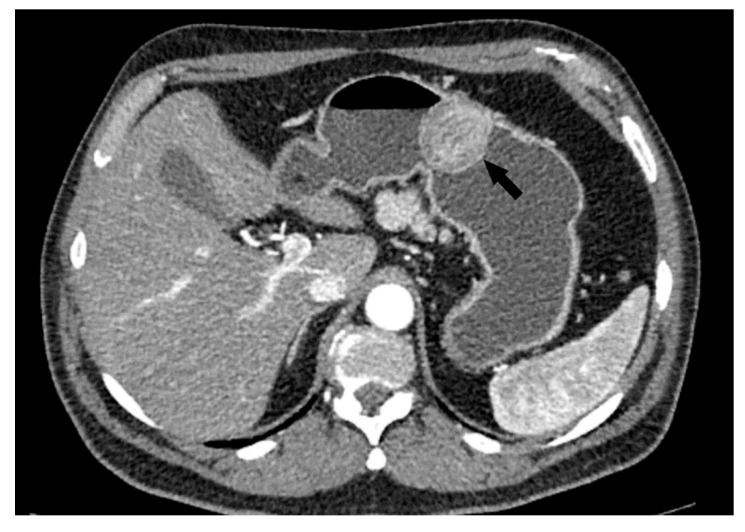
Endophytic growth of oval, solid GIST covered by intact mucosa (black arrow) in the antral region of the stomach.

**Figure 6:**
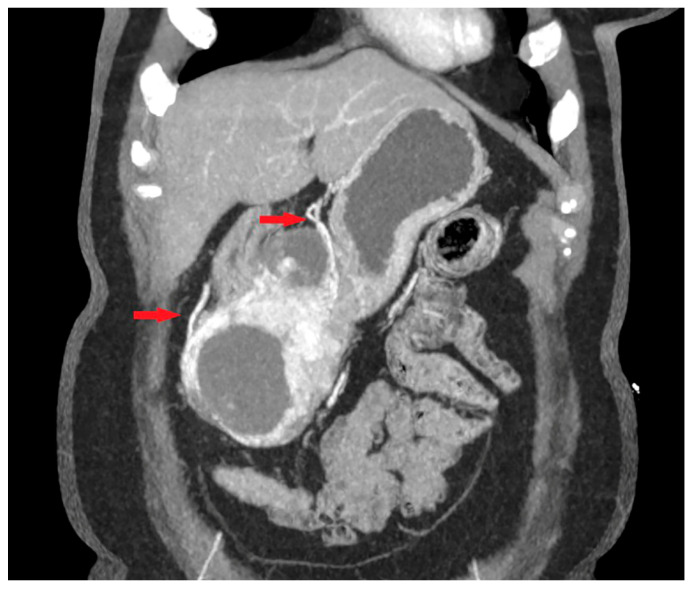
CT coronal view shows irregular, partially cystic tumor with hyperdense solid component and presence of peri- and intra-tumoral vascular vessels (red arrows) (EFDV).

**Figure 7:**
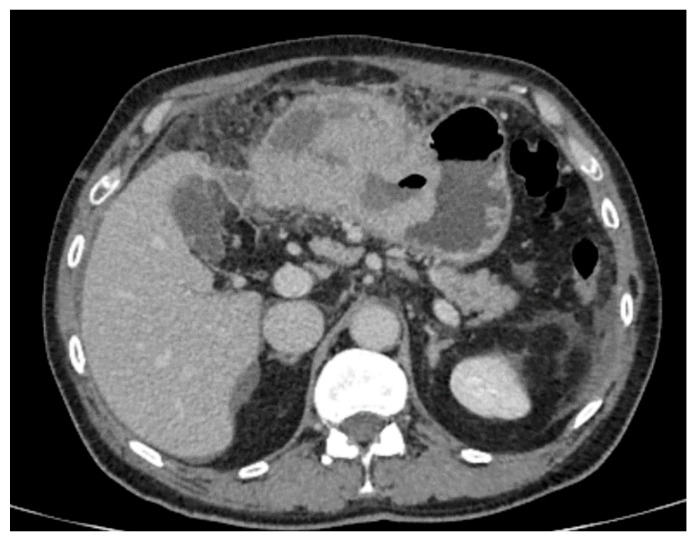
CT scan demonstrates massive necrotic tumor with ill-defined margins and disrupted mucosa with exulceration (HR GIST).

The following morphological characteristics of the tumor were analyzed:

1. Maximum diameter: the largest diameter of the tumor in mm (Figure 1);
2. Appearance of mucosa: intact/continuous and discontinuous (Figure 1 and Figure 2);
3. Tumor structure: solid–necrotic and cystic changes (Figure 1, Figure 2, Figure 3 and Figure 4);
4. Tumor shape: regular or irregular (Figure 1);
5. Tumor localization in relation to the region of the stomach: corpus, antrum and pylorus (Figure 1, Figure 2 and Figure 5);
6. Growth mode: exophytic/mixed and endophytic (Figure 1);
7. Level of opacification of the solid part of the tumor: weak and intense (Figure 3 and Figure 6);
8. The presence of visible enlarged vascular structures draining/feeding the tumor (EFDV “enlarged feeding or draining vessel”) (Figure 6);
9. Margin appearance: well-defined and ill-defined (Figure 1 and Figure 7).

### 2.4. CT Texture Analysis

**Figure 8:**
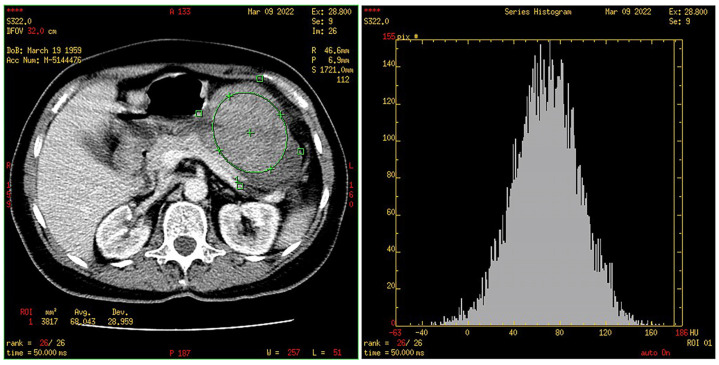
Segmented tumor (green ROI) with histogram.

**Figure 9:**
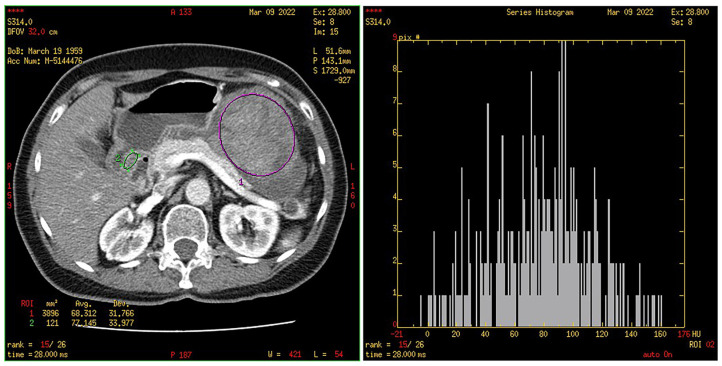
Segmented healthy stomach wall (green ROI) with histogram. Segmented tumor (purple ROI).

Texture analysis was performed with the software MaZda (Version 4.6 for Windows, Institute of Electronics, Technical University of Lodz, Poland). The solid part of the tumor was segmented into three consecutive sections in the portal venous phase. A healthy structure was also marked; in our case, it was a normal gastric wall. The values of the first-order texture, i.e., histogram parameters, were automatically obtained and were as follows: the normalized frequency of pixels of the lowest intensity (“min norm”) and the highest intensity (“max norm”), mean intensity (“mean intensity”) and standard deviation (“variance”), as well as “skewness “, i.e., asymmetry, and “kurtosis”, i.e., the peak/flatness of the histogram. The mentioned values were obtained for each of the three sections, while their mean values for the GISTs and stomach wall were used for further statistical data processing (Figure 8 and Figure 9).

### 2.5. Pathological Analysis and Risk Stratification of Gastric GISTs

The main therapeutic option for localized GISTs is surgery. Wedge resection and subtotal and total gastrectomy are the most frequently used surgical procedures. The resected tumor needs a complete pathohistological evaluation according to established protocols of fixation in 10% formaldehyde and incorporation in paraffin and hematoxylin and eosin. The presence of spindle or epithelioid cells or both is necessary for GIST definition as well as positive immunohistochemical staining for C-KIT or DOG-1. The TNM classification is the standard for risk stratification [21]. Miettinen et al. have established a classification system, AFIP classification, where tumor diameter, mitotic index and localization are the most significant prognostic factors [10]. In addition to metastatic risk, the grade of these tumors is determined by the value of the mitotic index, with a cut-off of five or fewer mitoses visualized per 5 mm^2^ or per 50 HPF. Based on these classifications, GISTs are further categorized into four different stages according to mitotic index and the size and presence of metastases in the lymph nodes, liver and peritoneum. We subclassified GIST patients into high-risk (HR GIST) (high-risk and intermediate-risk) and LR GIST (low-risk) groups.

### 2.6. Statistical Analysis

Normality of distribution of numerical data was assessed by the Kolmogorov–Smirnov test. Mean ± standard deviation (SD) or median value with range were presented depending on the distribution. The chi-square test or Fisher’s exact probability test were used to assess differences in morphological features between HR and LR GISTs and, for quantitative parameters, *t*-test of independent samples or the Mann–Whitney test were used depending on the normality of distribution. t-test for paired samples or Wilcoxon’s test for equivalent pairs were used in testing the differences in histogram texture parameters of gastric GISTs in comparison to the normal gastric wall. Univariate and multivariate binary logistic regression analysis was used to identify the morphologic characteristics and histogram texture parameters that are significant predictors of HR GISTs as well as to build a preoperative predictive model suggesting HR GISTs that was further tested by ROC analysis. The level of statistical significance was set at 0.05, while all statistical analyses were performed using SPSS software (Version 17.0 for Windows; SPSS, Chicago, IL, USA).

## 3. Results

The study included 79 patients with gastric GISTs (45 male, 34 female, with mean age 65 ± 11). HR GISTs were confirmed in 36 patients and LR GISTs in 43. In terms of age, there was no significant difference between the LR and HR groups (64 ± 12 vs. 62 ± 10, *p* = 0.772), nor was there a difference in terms of gender (26 vs. 19 men and 17 vs. 17 women, *p* = 0.472).

### 3.1. Tumor Diameter in HR and LR Group

**Figure 10:**
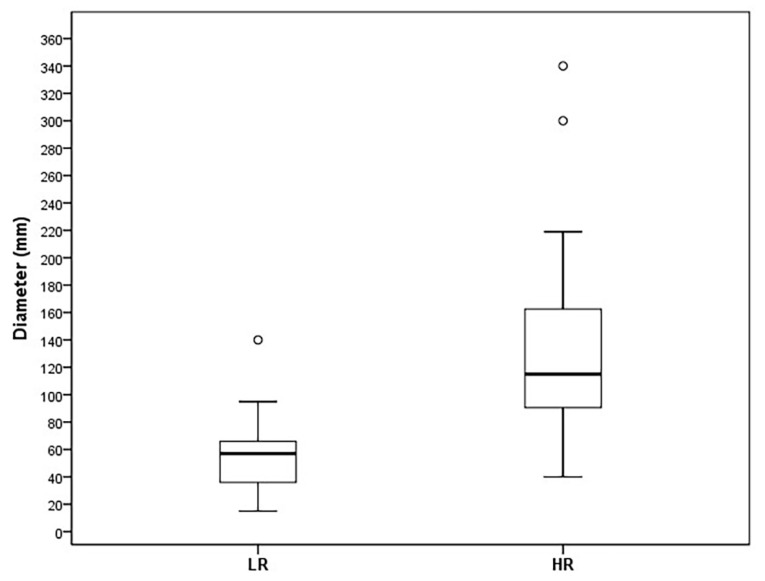
Box plot of tumor diameter (mm) in LR and HR group.

The smallest tumor diameter in the LR group was 15 mm and the largest was 150 mm (mean 56 ± 25 mm), while the range in the HR group was from 40 mm to 340 mm (mean 131 ± 58 mm), *p* < 0.001 (Figure 10).

**Figure 11:**
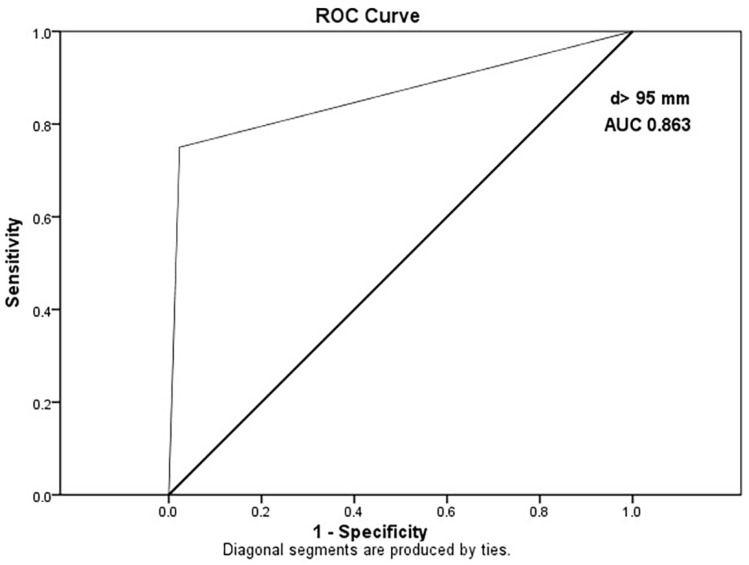
ROC curve representing a diameter cut-off value of 95 mm between the LR and HR group with an AUC of 0.863 (CI 0.772–0.954).

ROC analysis showed that a cut-off diameter of 95 mm most accurately predicted HR GISTs (AUC 0.863; CI 0.772–0.954), with a sensitivity of 98% and specificity of 75% (Figure 11).

Only one of forty-three LR GISTs was larger than 95 mm, but nine of thirty-six HR GISTs (25%) were smaller than 95 mm in maximal diameter.

### 3.2. Classic CT Features in HR and LR Group

A comparison of classic CT features of gastric GISTs in the HR and LR groups is presented in Table 1.

### 3.3. Histogram Parameters in HR and LR Group

The differences in textural parameters between HR and LR GISTs are shown in Table 2.

### 3.4. Histogram Parameters in HR and LR Group

The differences in textural parameters between HR and LR GISTs are shown in Table 3.

### 3.5. Univariate and Multivariate Predictive Models

**Figure 12:**
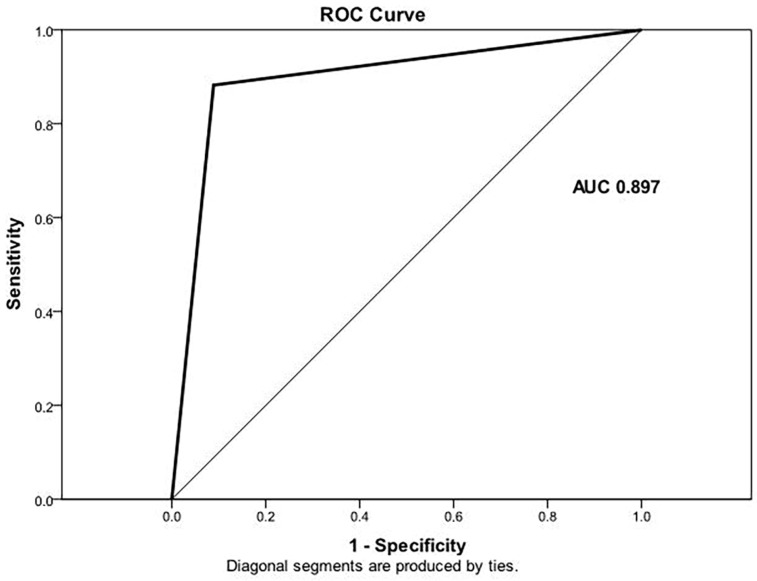
ROC curve shows the following linear regression model including diameter, margins, growth, shape, structure, mucosa, EFDV and max norm predicts HR GISTs with an AUC of 0.897 (CI: 0.817–0.976) with a sensitivity of 83.3%, specificity of 90.7% and accuracy of 87.3%.

Univariate regression analysis confirmed tumor diameter, margin appearance, growth pattern, lesion shape, structure, mucosal continuity, presence of EFDV and the textural parameter max norm as significant predictive factors for HR GISTs (Table 4) (Figure 12).

Multivariate regression analysis identified interrupted mucosa (*p* < 0.001) and presence of EFDV (*p* < 0.001) as independent predictive CT features for HR GISTs (Table 5).

**Figure 13:**
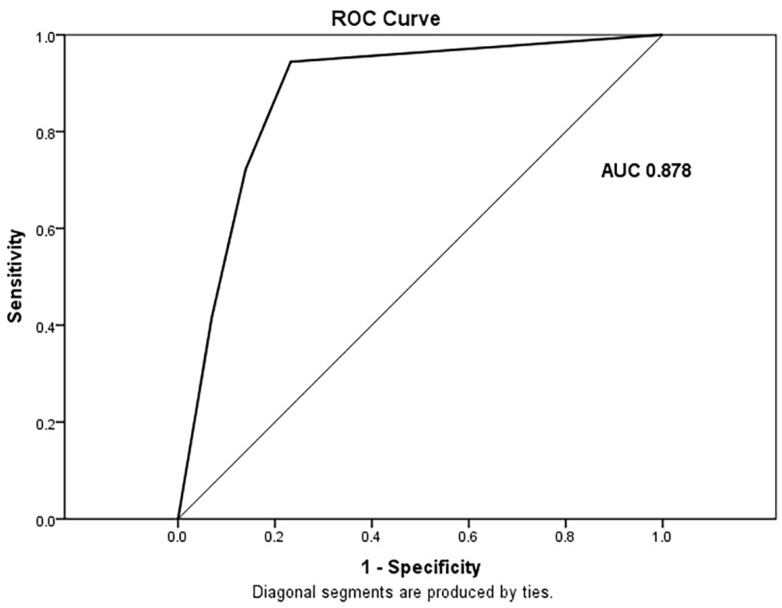
ROC curve of multivariate regression model with two independent predictors for HR GISTs (P (0–1)) = 0.346 × mucosa (1—continuous/2—interrupted) + 0.493 × EFDV (1—present/0—absent)–0.222). AUC = 0.878 (CI: 0.797–0.959), sensitivity 94%, specificity 77% and accuracy 88%.

ROC analysis showed the multivariate linear regression model with extracted mucosa appearance and presence of EFDV achieved an AUC of 0.878 (CI: 0.797–0.959) with a sensitivity of 94%, specificity of 77% and accuracy of 88% in the prediction of HR GISTs (Figure 13).

## 4. Discussion

Our study confirmed the great importance of morphological CT characteristics of GISTs, which proved to be significant predictive factors in the risk stratification of these tumors. Parameters such as diameter, localization, margins, growth pattern, structure, intensity of postcontrast tumor opacification, shape, continuity of the mucosa and the presence of EFDV showed statistical significance in the prediction of HR GISTs (Table 1). In our research, risk assessment was based on the TNM and AFIP calcification systems, where the diameter of the lesion is an important factor in predicting the metastatic risk of these tumors. A cut-off value of 5 cm has been established within many classifications, and lesions below 5 cm are considered benign variants of this tumor [23]. We also concluded that tumor diameter is a very important predictive factor of high metastatic potential in these tumors, with a cut-off value of 9.5 cm between the LR and HR groups. However, 25% of HR tumors in our series were smaller than 95 mm.

According to our results, the most common localization of GISTs was the area of the corpus and antrum, which coincides with the predominance of Cajal cells in the stomach wall in this area, which is consistent with the results of other studies [24]. Ill-defined tumor margins showed a high statistical significance in predicting HR GISTs in many previous studies [14,16]. Similarly, in our study, univariate regression analysis revealed that this parameter was a significant predictive factor in metastatic risk stratification. In the current study, growth patterns were observed to be both exophytic and endophytic, but also a combination of both variants. Thirty-five patients with proven HR GISTs showed an exophytic and mixed growth pattern. This growth pattern was proven to be a highly statistically significant parameter regarding HR GISTs, which was also shown in other studies as well. Peng et al. used multivariate regression analysis and identified exophytic growth, irregular shape and discontinuous gastric mucosa covering the tumor as significant and independent predictors of HR GISTs [25]. In a series of 129 patients, Zhou et al. analyzed the morphological characteristics of tumors and their regression model extracted tumor diameter, mixed tumor growth and the presence of EFDV as independent predictive factors of high-risk GISTs [19]. When the intensity of postcontrast opacification was analyzed, the largest number of patients (62) showed lower postcontrast enhancement in the portal venous phase of the examination. Among them, 33 patients had a HR GIST. In the present study, regular tumor shape (oval and round shape) was found mostly in LR GISTs, while an irregular, lobulated CT tumor presentation correlated with a higher metastatic risk. An irregular tumor shape is certainly a very important and statistically significant parameter in the prediction of HR GISTs. In our study, the linear regression model included this morphological CT feature as a predictive factor for HR GISTs (AUC 0.897). In previous studies, irregular tumor shape was exclusively characteristic of HR GISTs [18,26]. The structure of tumors can vary from homogeneous and predominantly solid to heterogeneous due to the appearance of intralesional necrosis and cystic degeneration. CT examination clearly shows the mentioned structural differences. Solid and partially necrotic lesions are predominant within the LR group, while cystically degraded tumors were mostly high-risk. Contrary to our results, previous studies have shown that necrotic tumors are associated with a higher mitotic index and metastatic risk. A high MI reflects more intense tissue proliferation, which results in structural degradation and the appearance of intratumor hemorrhage, necrosis and cystic degeneration. Therefore, it is likely that the necrosis and heterogeneity of a tumor observed by visual inspection could be associated with an increased number of mitoses. Larger lesions tend to have a heterogeneous structure and correspond to high-risk tumors. In a study by Grazzini et al., necrosis was shown to be an independent predictive factor for HR GISTs [15]. GISTs are tumors of submucosal localization. In smaller lesions (low-risk GISTs), the mucosa is usually smooth and continuous. Interruption of the continuity of the mucosa leads to the formation of ulcers or umbilications, which are often the source of bleeding. In our study, discontinuous mucosa is a statistically significant factor in the prediction of HR GISTs, in line with the results of previous studies [20]. The multivariate linear regression model in the present study included mucosa appearance and the presence of EFDV as significant predictive parameters of HR GISTs (AUC 0.878). Presence of EFDV was an independent predictor of HR GISTs. This parameter is a reliable index for evaluating the malignancy of these tumors, which can be explained by the fact that accentuated neovascularization is crucial in tumor proliferation and the occurrence of distant hematogenous metastases. This result is consistent with the results of other studies underlining this parameter as an important predictor of high-risk tumors [24,27]. CTTA of tumor heterogeneity showed a significant contribution in the characterization of lesions, such as distinguishing benign from malignant tumors or indicating more biologically aggressive lesions. This technique has also shown progress in the initial evaluation of tumors before treatment and in evaluating the therapeutic response for some types of tumors as well [19]. Although there are encouraging data suggesting that CTTA is a promising imaging biomarker, one should not forget the significant variability in methods and examined parameters and in association with biological correlates. Before this advanced CT diagnostic method can be considered for global clinical practice implementation, the standardization of tumor segmentation and measurement techniques, as well as postprocessing, is necessary to identify the most important textural parameters. The continuation of research, external verification of histopathological correlates and a specified, uniform formulation of reports are also of great importance for the further application of this method [19]. Tumors are generally heterogeneous lesions not only at the cellular level, but also genetically and phenotypically, with spatial heterogeneity of cell density, angiogenesis and necrosis. This tissue heterogeneity is an important factor that has an impact on prognosis and treatment, bearing in mind that more intense structural degradation of the lesion and its heterogeneity can be associated with very malignant and aggressive tumor behavior with increased resistance to treatment [25]. CTTA is only one segment of the growing and very promising field of radiomics, which involves the extraction, complex analysis and interpretation of quantitative parameters obtained from diagnostic images. In our study, histogram parameters were analyzed in 79 patients and max norm showed statistical significance in terms of predicting HR GISTs. In contrast, a study by Choi et al. including 145 GIST patients showed that the main predictive factors for HR GISTs were kurtosis and MPP (mean positive pixels) [17]. In the same study, in the HR GIST group, lower mean, SD and MPP values were observed, while the kurtosis parameter was significantly higher. Moreover, higher values of skewness and kurtosis were characteristic of lesions with a high mitotic index. Based on subjective assessment, lesion characteristics such as lower density, necrosis and mucosal ulceration were identified as predictive factors for HR GISTs [17]. It should be kept in mind that histogram parameters may have a different significance depending on the type of tumor, the type of imaging performed as well as the analytical method. A high mitotic index in GISTs reflects rapid tissue proliferation that leads to a heterogeneous structure and necrotic and cystic degradation of lesions, so it can be concluded that visual confirmation of damaged tumor tissue may suggest a higher mitotic index. Previous studies have shown a correlation of larger diameter (>11 cm), tumor heterogeneity and presence of necrosis with a higher mitotic index and metastatic risk [14,27]. In addition, necrotic lesions showed low mean and MPP values and higher values for kurtosis, which is consistent with the results of our research. This can be explained by the low attenuation caused by tissue necrosis and increased heterogeneity of the tumor structure [28]. In a study by Liu et al., in terms of predicting the metastatic risk of GISTs, it was shown that the peak value on the histogram (maximum frequency) has the greatest superiority in comparison with other parameters of texture analysis, which is in concordance with our results [29]. Another study by the same authors indicated a correlation between CT texture parameters and immunohistochemical biomarkers such as E-cadherin, Ki67, VEGFR2 and EGFR in 139 patients with gastric cancer [30].

Our study has several limitations. The sample of patients was relatively small and our research did not include a follow-up of the included patients. Certainly, a prospective study with a larger cohort is needed in further research to confirm the findings of this study and to incorporate the analyzed diagnostic method into daily clinical practice.

## 5. Conclusions

Our study resulted in a regression model that identified mucosal discontinuity and the presence of EFDV features as independent and significant predictors of HR GISTs, which leads us to the conclusion that morphological CT features have the greatest value in the non-invasive, preoperative prediction of metastatic risk of gastric GISTs. A significant statistical significance was shown regarding the functional parameter max norm within the textural analysis of these tumors. The incorporation of advanced CT techniques into the basic diagnostic protocol can further benefit the preoperative assessment of risk stratification in GISTs. Preoperative risk stratification is of great significance to evaluate the risk of tumor recurrence and guide treatment planning before and after surgery. This improves the management of treatment, especially in terms of the application of neoadjuvant therapy, which further enables tumor shrinkage, reduces tumor ruptures, increases overall survival rates and optimizes surgical resection and systemic control of the disease. Our model may serve as a diagnostic tool for the non-invasive prediction of HR GISTs to support personalized treatment strategies.

## Figures and Tables

**Table 1:** Comparison of classic CT features of gastric GISTs in the HR and LR groups.

CT Characteristics of Gastric GISTs		LR GISTs (*n* = 43)	HR GISTs (*n* = 36)	*p* Values
Localization	Body	13	21	0.014
	Antrum	23	8	
	Pylorus	7	7	
Margins	1—well defined	42	28	0.006
	2—ill defined	1	8	
Growth pattern	1—exophytic/mixed	32	35	0.005
	2—endophytic	11	1	
Tumor enhancement	0—low	29	33	0.009
	1—high	14	3	
Shape	1—regular (round)	38	11	<0.001
	2—irregular	5	25	
Structure	1—solid/necrotic	34	10	<0.001
	2—cystic	9	26	
Mucosa	1—continuous	36	13	<0.001
	2—discontinuous (rupture)	7	23	
EFDV *	0—absent	37	10	<0.001
	1—present	6	26	

* Enlarged feeding or draining vessels.

**Table 2:** Histogram parameters in the LR and HR groups. *: statistically significant parameter. Bold: a significant result.

Histogram Parameters	LR GISTs (*n* = 43)	HR GISTs (*n* = 36)	*p*
Min norm	32,866.776 (32,816.739–33,866.283)	32,851.065 (32,815.016–33,875.819)	0.032 *
Max norm	612.419 (230.676–1572.068)	524.927 (177.284–835.740)	0.052
Mean	−0.058 (−3.570–0.213)	−0.001 (−0.428–0.304)	0.093
Variance	−0.113 (−0.560–8.145)	−0.096 (−0.557–2.248)	0.806
Skewness	32,812.667 (32,709.333–33,815.667)	32,800.166 (32,764.000–33,813.667)	0.182
Kurtosis	32,837 (32,788.000–33,841.333)	32,822.166 (32,785.667–33,838.667)	0.058

**Table 3:** Histogram parameters in the LR and HR GIST groups. *: statistically signifikant parameter; **: highly statistically significant parameter.

Histogram Parameters	LR GISTs (MI ≤ 5) (*n* = 52)	HR GISTs (MI > 5) (*n* = 27)	*p*
Min norm	32,867.360 (32,816.740–33,875.819)	32,845.770 (32,815.060–33,844.490)	0.007 **
Max norm	610.350 (230.700–1572.100)	516.245 (177.280–835.730)	0.051
Mean	−0.051 (−3.570–0.304)	0.007 (−0.428–0.290)	0.089
Variance	−0.106 (−0.560–8.145)	−0.113 (−0.557–2.248)	0.836
Skewness	32,815.160 (32,709.300–33,815.600)	32,797.000 (32,764.000–33,813.000)	0.035 *
Kurtosis	32,838.000 (32,788.000–33,841.000)	32,818.670 (32,785.660–33,827.330)	0.009 **

**Table 4:** Classical CT morphological and histogram predictive factors for HR GISTs obtained by univariate regression analysis.

Variables in the Equation							
		B	S.E.	Wald	df	Sig.	Exp (B)
Step 1	Diameter (mm)	0.013	0.013	0.936	1	0.333	1.013
	Margins	−0.120	1.502	0.006	1	0.936	0.887
	Growth pattern	−2.425	1.570	2.386	1	0.122	0.088
	Shape	1.566	0.961	2.653	1	0.103	4.786
	Structure	0.554	0.987	0.315	1	0.575	1.740
	Mucosa	2.219	0.942	5.551	1	0.018	9.199
	EFDV	2.067	0.961	4.628	1	0.031	7.903
	Max norm	−0.001	0.002	0.398	1	0.528	0.999
	Constant	−4.751	2.994	2.518	1	0.113	0.009

**Table 5:** Significant predictive parameters of HR GISTs by multivariate logistic regression analysis.

Model		Unstandardized Coefficients		Standardized Coefficients	t	Sig.
		B	Std. Error	Beta		
1	(Constant)	−0.222	0.127		−1.742	0.086
	**Mucosa** 1—continuous 2—ruptured	0.346	0.092	0.337	3.779	0.000
	**EFD** 0—absent 1—present	0.493	0.091	0.486	5.445	0.000

## Data Availability

Data are contained within the article.
